# Utility of Fundus Autofluorescence and Optical Coherence Tomography in Measuring Retinal Vascular Thickness, Macular Density, and Ophthalmic Manifestations in Women with Gestational Diabetes Mellitus

**DOI:** 10.3390/life14121596

**Published:** 2024-12-03

**Authors:** Rami Al-Dwairi, Omar Altal, Marwa Fares, Sharaf H. Adi, Shahed A. Said, Asmaa Shurair, Rania Al-Bataineh, Ihsan Aljarrah, Seren Al Beiruti, Ahmed H. Al Sharie, Abdelwahab Aleshawi

**Affiliations:** 1 Department of Special Surgery, Division of Ophthalmology, Faculty of Medicine, Jordan University of Science & Technology, Irbid 22110, Jordan; marwa11kf@gmail.com (M.F.); dr.sharafadi@gmail.com (S.H.A.); asmaashurair@gmail.com (A.S.); seren.beiruti@yahoo.com (S.A.B.); 2 Department of Obstetrics and Gynecology, Faculty of Medicine, Jordan University of Science & Technology, Irbid 22110, Jordan; altal_omar@yahoo.com (O.A.); shahdazmii2018@gmail.com (S.A.S.); 3 Department of Family Medicine, Faculty of Medicine, Jordan University of Science & Technology, Irbid 22110, Jordan; raniabataineh123@gmail.com; 4 Thin Films and Nanotechnology Lab, Department of Physics, Jordan University of Science & Technology, Irbid 22110, Jordan; ihsanaljarrah@gmail.com; 5 Department of Pathology and Microbiology, Faculty of Medicine, Jordan University of Science & Technology, Irbid 22110, Jordan; ahmedalsharie3@gmail.com

**Keywords:** gestational diabetes, diabetic retinopathy, OCT, OGTT

## Abstract

**Background:** Gestational diabetes mellitus (GDM) is a transient elevation of blood glucose during pregnancy. It is typically not associated with diabetic retinopathy. However, certain investigators revealed retinal microvascular injury. In this study, we aimed to assess the ophthalmic findings, optical coherence tomography (OCT) parameters, and retinal vascular thickness and macular density through fundus autofluorescence (FAF). **Methods**: Prospectively, women diagnosed with GDM were enrolled in this study. All the participants underwent comprehensive ophthalmic examination. Furthermore, macular OCT with analysis of the central subfield thickness (CST) and total thickness was carried out. Moreover, FAF was performed, and the macular density and retinal vascular thickness were extracted using ImageJ software. **Results**: Thirty-four women were enrolled. The mean maternal age was 32.7 years. No participant had diabetic retinopathy, nine eyes had early cataract, and two eyes had keratoconus. Higher levels for the 1 h oral glucose tolerance test (OGTT) were associated with a drop in the CST and total thickness. Moreover, women who underwent CS had higher levels of total thickness. Higher levels for the fasting OGTT were associated with a thinner inferior temporal retinal artery. Pregnant women with miscarriages had lower macular density on FAF, as represented by lower values of integrated density and mean gray values. Higher levels for the fasting OGTT were associated with higher values of integrated density. **Conclusions**: Although GDM is typically not associated with diabetic retinopathy, microscopic changes involving the microvascular environment and the macula may occur. Regular ophthalmic screening for women with GDM may be advised. Larger studies with more investigations may reveal further findings.

## 1. Introduction

Gestational diabetes mellitus (GDM) is one of the most common medical complications of pregnancy. It is characterized by the onset of hyperglycemia or impaired glucose tolerance during gestation, typically diagnosed between 24 and 28 weeks of pregnancy. Unlike other forms of diabetes, GDM usually resolves postpartum [1,2]. Numerous risk factors for GDM have been identified, including a history of GDM in a previous pregnancy, macrosomia, congenital anomalies, neonatal death, low educational status, smoking, a body mass index (BMI) ≥ 25, a family history of type 2 diabetes mellitus, and pregnancy-induced hypertension [1,3].

Although GDM typically resolves postpartum, it is associated with significant short- and long-term complications for both the mother and the fetus. The maternal complications include an increased likelihood of emergency cesarean delivery and preeclampsia, while the fetal complications may include macrosomia, neonatal hypoglycemia, birth asphyxia, shoulder dystocia, respiratory distress syndrome, and hyperbilirubinemia [4]. Additionally, GDM has been linked to an increased long-term risk of obesity, cardiovascular disease, and glucose intolerance, which may progress to type 2 diabetes in both the mother and her offspring [5]. Within 10 years postpartum, approximately 50% of women with a history of GDM develop type 2 diabetes [6].

Pregnancy induces a range of multi-organ changes, including alterations in the eyes. Some of these changes may exacerbate pre-existing conditions, while others may arise as a result of normal physiological processes or pregnancy-related complications [7]. Physiological changes during pregnancy, including metabolic, immunologic, vascular, and hormonal fluctuations, have been shown to exacerbate diabetic retinopathy, which, if left untreated, can lead to permanent vision loss [8]. GDM, as a common pregnancy complication, is associated with an increased incidence of pathological ophthalmic conditions. Specifically, the incidence of glaucoma, retinal detachment, and DR is significantly higher in GDM patients [6]. However, conflicting data exist in the literature regarding the necessity of including GDM patients in diabetic retinopathy screening [6,8], highlighting the need for further research to address policy changes concerning this matter.

This prospective observational study aims to investigate the ocular parameter changes in GDM patients. Specifically, we will discuss the effects of various gestational variables and optical parameters on optical coherence tomography (OCT), the retinal vascular thickness, and the macular density on fundus autofluorescence (FAF).

## 2. Methods

### 2.1. Participants and Data

This study was conducted at King Abdullah University Hospital, a tertiary clinical and research center affiliated with Jordan University of Science and Technology, during the period of June 2023 to February 2024. Prospectively and after obtaining the approval of the institutional review board, all pregnant women diagnosed GDM were invited to participate in this study, which aimed to investigate the ophthalmic findings in these women and to assess the OCT and FAF parameters in this group of patients. Electronic databases and interviews were utilized to collect data from the participants. Demographic data and the medical history were obtained. In addition, a detailed maternal and obstetric history was obtained from the participants. Furthermore, a comprehensive ophthalmic assessment and history were performed.

All pregnant women with GDM aged from 20 to 45 years were enrolled in this study. Females with singleton pregnancy between 20 and 33 weeks of gestation were included. The exclusion criteria included a chronic diabetes history prior to gestation, high blood pressure, a positive oral glucose tolerance test (OGTT) before 26–28 weeks of pregnancy, and albuminuria in pregnant women. Moreover, participants with renal or cardiorespiratory ailments and who were previously taking any hypoglycemic agents were excluded. Furthermore, a contraindication for metformin, a fetal anomaly on ultrasound examination, gestational hypertension, preeclampsia, fetal growth restriction (birth weight <10th centile for its gestational age on ultrasound comparing with a 1st dating or early 2nd trimester scan), and ruptured membranes were included in the exclusion list. Lastly, any women with ocular diseases that adversely affect the vision were excluded.

This study was carried out in compliance with the ethical guidelines in place at our institute, taking the Declaration of Helsinki as an ethical guideline for research involving human subjects. Written informed consent was obtained from all the participants.

### 2.2. Obstetrics Settings

The course and care of pregnancy was the responsibility of a single consultant in maternal–fetal obstetrical medicine. GDM was definition by the oral glucose tolerance test according to the World Health Organization criteria: using a 75 g OGTT after overnight fasting (8 to 10 h) at 26–28 weeks gestation. Diagnosis of GDM was made with at least two elevated readings for the OGTT, fasting glucose (>5.3 mmol/L), at one hour (10 mmol/L) and at two hours (8.6 mmol/L). These pregnant women were assessed for high risk factors, including a family history of diabetes, previous history of macrosomia, previous history of GDM, and previous history of poor obstetric outcome (miscarriage, intrauterine fetal death). Women who screened negative at the first antenatal visit had repeat screening at 28, 32 and 36 weeks of pregnancy.

The treatment commenced with similar guidelines. Metformin therapy was started at a dose of 500 mg once or twice daily with food and increased, typically over a period of 1 to 2 weeks, in intermittent doses dependent upon the glycemic control of the patient. If the targets were not achieved with metformin and dietary modification alone, subcutaneous insulin was commenced. Patients were asked to measure their blood sugar levels daily and note them in a diary, and after every week, their doses of metformin and insulin were adjusted according to the control of their blood sugar levels.

The mode of delivery depended on the antenatal course, the presence of antenatal complications, the experience of the obstetricians, and the choice of the pregnant women. The birth weight of the baby was calculated immediately after birth and was presented in grams.

### 2.3. Ophthalmological Examination Settings

All the patients underwent comprehensive ophthalmic examinations by a vitreoretinal consultant or well-trained residents during the follow-up visits with the obstetrics team, including the best-corrected visual acuity (BCVA) determined using a Snellen visual acuity chart by decimal unit. Then, the BCVA was converted to the LogMAR visual acuity. A Goldmann applanation tonometer was utilized to measure the intraocular pressure. Slit-lamp and indirect biomicroscopes were used to assess the lens status, anterior segment, and fundus conditions. The pupil was dilated with tropicamide eyedrops.

### 2.4. OCT Measurements

**Figure 1:**
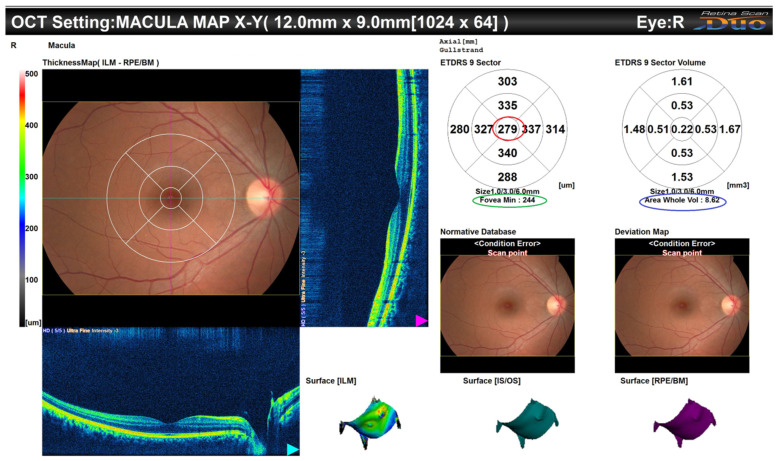
OCT of the macula of one of the participants, demonstrating the CST (in red circle), the foveal minimum (in green circle), and the volume of the whole area (in blue circle).

The acquisition of the OCT images was performed twice by two experienced optometrists to ensure the accuracy of the images. Then, the images were reviewed by a consultant vitreoretinal surgeon. Any image with low quality was repeated. Following the process of pupillary dilatation by tropicamide 1% eyedrops, each participant was seated in front of the OCT scanner and their head was stabilized on the chin rest. The macular area was analyzed using spectral domain (SD)-OCT (Retinascan RS-3000; NIDEK, Gamagori, Japan). Four radial scans, each measuring 6 mm in length, were positioned at the fovea in the macula at angles of 0°, 45°, 90°, and 135°. The scans comprised 1024 A-scans utilizing high-definition (50 HD) frame enhancement software. This equipment is equipped with a light source that emits electromagnetic radiation with a wavelength of 880 nm. The instrument had demonstrated excellent consistency and accuracy in measuring both healthy and diseased eyes. The Early Treatment Diabetic Retinopathy Study (ETDRS) was used to acquire nine consecutive macular sectors located in the foveal center [9]. Retinal thickness was determined by measuring the distance between two interfaces at each location along the x-axis of the scan. A low-intensity light source was utilized for internal fixation, and the procedure was performed in a dimly lit environment. Images exhibiting a signal strength below 7/10, inadequate centering, motion artifacts, and dark areas were removed, ensuring a minimum of 2 images of acceptable quality. The retinal thickness was defined as the distance between the vitreoretinal interface and the anterior surface of the retinal pigment epithelium along each A-scan. The central subfield thickness (CST) was the average thickness within a central 1 mm area from the inner limiting membrane to the retinal pigment epithelium layer. Other measurements performed in this study were the fovea minimum (representing the thinnest area at the fovea), total thickness (which is the summation of the thickness of the nine ETDRS areas), and total volume (which is the summation of the volume of the nine ETDRS areas). Figure 1 presents an example from one of the participant’s OCT.

### 2.5. Fundus Autofluorescence (FAF) Acquisition

The acquisition and analysis of the FAF images were performed twice by the same two optometrists to ensure the accuracy and consistency, and to minimize the interobserver variability. Then, the images were reviewed by a consultant vitreoretinal surgeon. FAF imaging of the macula was obtained using a confocal scanning laser ophthalmoscope (Spectralis HRA, Heidelberg Engineering, Heidelberg, Germany). A 30 × 30° area centered at the fovea was scanned using excitation with a 488 nm argon laser and 500 nm filter. An image resolution of 1536 × 1536 pixels was used. The gain level was adjusted to delineate the major vessels and the disc on a single scan image, followed by averaging for sufficient quality. Macula-centered and optic-disc-centered images were acquired. The automatic real-time (ART) averaging mode was chosen, and 100 frames were captured to maximize the signal-to-noise ratio.

### 2.6. Retinal Vessel Diameter and Macular Density Measurements

**Figure 2:**
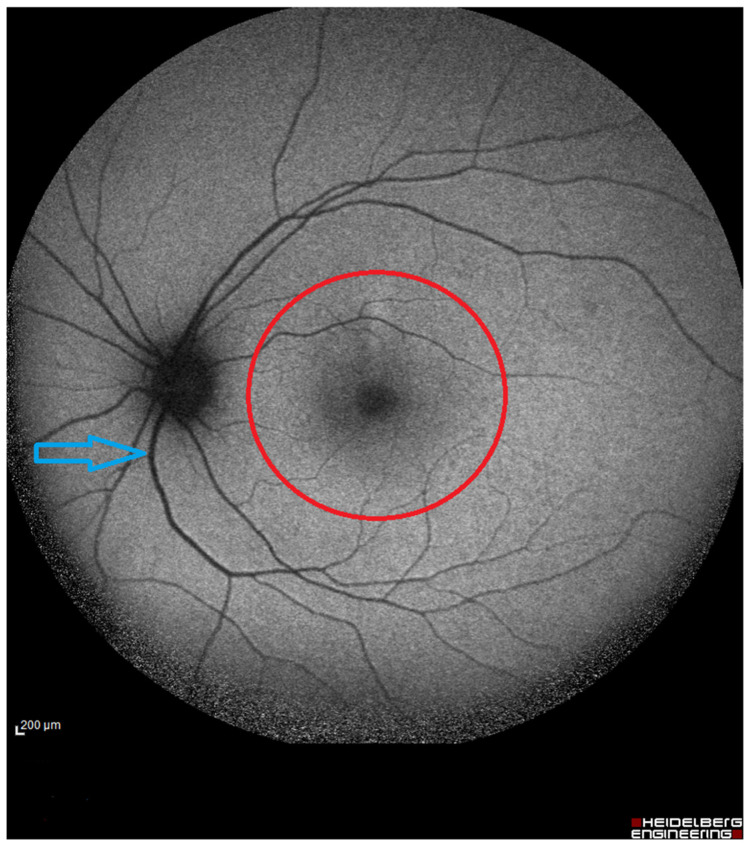
FAF of one of the participants showing the 5 mm macular area that was investigated for the density analysis. Also, it shows the place (in blue arrow) where the thickness of the major retinal vessels was measured (at 2 mm from the outer margin of the optic disc).

The analysis of the FAF images was conducted by an experienced investigator through ImageJ software 1.53K (National Institutes of Health, Bethesda, MD, USA). First, the superior-temporal retinal artery and vein diameter, and the inferior-temporal retinal artery and vein diameter, were measured. The thickness was measured for both eyes at the point 2 mm away from the margin of the optic disc using the straight ruler tool of the software. Figure 2 shows the points where the measurements of the retinal vessel diameter were calculated.

To quantitatively evaluate the FAF, the luminosity of the autofluorescence in a circle with a diameter of 5 mm at the center of the fovea was measured at each time point. Specifically, the average gray value of the autofluorescence in this area adjacent to the retinal vascular arcade was compared to that of the arcade vessels (veins). We quantified the mean signal intensity of 1000 pixels in the optic disc where the retinal pigmented epithelium and photoreceptors are absent as the zero point in individual images. This analysis allowed us to calculate the ratio of the autofluorescence intensity, providing a reliable indicator of the changes occurring in the treated areas. The following parameters were obtained: mean gray value, which is defined as the average gray value within the selection. This value is the summation of the gray values of all the pixels in the selection divided by the number of pixels. The minimum and maximum gray values are the minimum and maximum gray values within the selection, respectively. The integrated density is the summation of the values of the pixels in the image or selection and it is equivalent to the product of the area and mean gray value. The raw integrated density is the summation of the pixel values in the selected area. The following formulas summarize the measured parameters:

The raw integrated density = sum of pixel values in the selected area

The mean gray value = raw integrated density/(area in pixels)

The integrated density = raw integrated density × (area in scaled units)/(area in pixels)

### 2.7. Statistical Analyses

The collected data were entered into a spreadsheet and analyzed using the IBM SPSS statistical package for Windows v.29 (Armonk, NY, USA). Nominal variables were expressed as the frequency (percentage) and continuous variables as the mean ± standard error of the mean (SEM). Data normality was tested using the Kolmogorov–Smirnov test. The statistical significance between the study groups was determined by the ANOVA test for continuous variables. A simple linear regression test was utilized to assess the relation between two continuous variables. A statistically significant result was considered if *p* ≤ 0.05. The power of analysis equation was used to estimate the sample size with the assumption of the power of analysis at 90%, alpha level of 0.05, anticipated mean CST of 190 microns, and populational CST of 200 microns. The yielded required sample size was 51 eyes.

## 3. Results

### 3.1. General Demographic and Clinical Characteristics

A total of 34 pregnant women and 68 eyes were included in the analysis cohort. The mean maternal age was 32.7 ± 0.7 years, and the mean gestational age at delivery was 38.2 ± 0.2 weeks. The mean gestational age at the diagnosis of GDM was 30.3 ± 0.3 weeks, while the mean gestational age at the ophthalmic examination was 30.9 ± 0.7 weeks. The gestational parameters showed an average gravidity of 4.0 ± 0.2 and miscarriages of 0.88 ± 0.10. For GDM treatment, 5 participants (14.7%) were treated with diet only, 12 (35.3%) were treated with an oral hypoglycemic agent (OHGA)-based regimen, and 17 (50%) were treated with an insulin-based regimen. The mean values for the fasting, one-hour, and two-hour OGTT were 5.49 ± 0.10 mmol/L, 9.81 ± 0.30 mmol/L, and 8.38 ± 0.20 mmol/L, respectively. Regarding the mode of delivery, 28 participants (82.4%) delivered via cesarean section (CS), while 6 (17.6%) delivered through normal vaginal delivery (NVD). The cohort’s birth weight was 2763.70 ± 0.10 g.

Ophthalmic examination of the cohort revealed cataracts in nine eyes (13.2%); these cataractous changes were very mild and did not interfere with the OCT or FAF acquisition. Corneal and anterior segment pathology was observed in two eyes (2.9%) (keratoconus), and no cases of posterior segment pathology (no diabetic retinopathy). The optical parameters included the uncorrected visual acuity (0.81 ± 0.03 LogMAR), best-corrected visual acuity (0.97 ± 0.01 LogMAR), intra-ocular pressure (IOP) (14.9 ± 0.2 mmHg), and spherical equivalent (−1.55 ± 0.20 diopter). The OCT parameters included the CST, foveal minimum, total thickness, and total volume, with means of 259.70 ± 5.50 microns, 200.70 ± 6.10 microns, 2692.60 ± 32.80 microns, and 8.30 ± 0.10 mm^3^, respectively. Autofluorescence images were used to measure the retinal vascular thickness, with the following mean values: 0.1217 ± 0.003 mm for the superior-temporal retinal artery, 0.1507 ± 0.004 mm for the superior-temporal retinal vein, 0.1286 ± 0.003 mm for the inferior-temporal retinal artery, and 0.1535 ± 0.004 mm for the inferior-temporal retinal vein. Additionally, autofluorescence images were used to assess the macular density parameters, including the total area of investigation (28.06 ± 0.01 mm^2^), mean gray value (135.54 ± 3.10 pixels), maximum gray value (231.89 ± 3.90 pixels), minimum gray value (37.82 ± 4.70 pixels), integrated density (3789.47 ± 86.40 pixels/mm), and raw integrated density (7702068.87 ± 1784.88 pixels/mm). Table 1 summarizes the general demographic and clinical characteristics of the study cohort.

### 3.2. Factors Affecting OCT Parameters

In examining the factors affecting the OCT parameters (i.e., CST and total thickness), none of the following showed a significant association: maternal age, gestational age at delivery, gestational age at ophthalmic examination, gestational parameters, method of GDM treatment, or fetal weight. Regarding the mean values for the OGTT, only the one-hour OGTT resulted in a significant decrease in the regression coefficient (B) in both the CST (−7.28 ± 3.10 microns, *p* = 0.024) and total thickness (−54.57 ± 17.20microns, *p* = 0.030), whereas the fasting and two-hour OGTT had no significant effect. As for the mode of delivery, no significant effects were found on the CST; however, the mean total thickness in patients who underwent CS were significantly higher than those who had an NVD (2763.47 ± 236.40 microns vs. 2395.10 ± 346.10 microns, *p* = 0.001). A statistically significant negative relationship was observed between the best-corrected visual acuity and CST (regression coefficient (B) = −277.27 ± 11.80 microns, *p* = 0.018), while no significant effect was observed on the total thickness. The IOP was not significantly associated with either the CST or total thickness. Lastly, a statistically significant negative association was found between the spherical equivalent and the total thickness (regression coefficient (B) = −97.05 ± 14.60 microns, *p =* 0.032), with no effect on the CST. Table 2 summarizes the factors affecting the OCT parameters.

### 3.3. Factors Affecting Thickness of Retinal Vasculature

Our analysis of the association between several variables and the retinal vasculature thickness (i.e., superior-temporal retinal artery, superior-temporal retinal vein, inferior-temporal retinal artery, and inferior-temporal retinal vein) revealed several significant relationships. A statistically significant negative association was observed between the fasting OGTT and the inferior-temporal retinal artery thickness (regression coefficient (B) = −0.010 ± 0.004 mm, *p* = 0.010). The mode of delivery yielded a significantly thicker superior-temporal retinal vein in those who delivered via CS compared to those who had an NVD (0.155 ± 0.02 mm vs. 0.128 ± 0.03 mm, *p* = 0.018). Among the optical parameters, the best-corrected visual acuity was associated with a significant decrease in both the inferior-temporal retinal artery and vein thickness, with regression coefficients (B) of −0.160 ± 0.07 mm (*p* = 0.030) and −0.233 ± 0.09 mm (*p* = 0.020), respectively. In contrast, neither maternal age, gestational age at delivery, gestational age at ophthalmic examination, gestational parameters, methods of treating GDM, nor fetal weight yielded significance. Table 3 summarizes the factors affecting the thickness of the retinal vasculature.

### 3.4. Factors Affecting Density Parameters of Autofluorescence Images

The examination of the relationship between several variables and the macular density parameters through autofluorescence images (i.e., mean gray value and integrated density) demonstrated several significant effects. Specifically, miscarriages, out of the gestational parameters, affected both density parameters, with regression coefficients (B) of −6.50 ± 3.10 pixels for the mean gray value (*p* = 0.042) and −173.75 ± 58.70 pixels/mm for the integrated density (*p* = 0.049). Moreover, the fasting OGTT was the only OGTT parameter to have a significant effect, with a significant increase in the integrated density (228.00 ± 89.40 pixels/mm, *p* = 0.040). Regarding the optical parameters, the spherical equivalent significantly affected both macular density parameters, with regression coefficients (B) of −10.95 ± 2.90 pixels for the mean gray value (*p* = 0.002) and −298.90 ± 81.30 pixels/mm for the integrated density (*p* = 0.001). The other variables (i.e., maternal age, gestational age at delivery, gestational age at ophthalmic examination, methods of GDM treatment, mode of delivery, and fetal weight) lacked significance in relation to the macular density parameters. Table 4 summarizes the factors affecting the density parameters of the autofluorescence images.

## 4. Discussion

To the best of our knowledge, this is the first study conducted to evaluate the clinical ophthalmic manifestations, macular OCT analyses, retinal vascular thickness, and FAF macular density analyses in pregnant women with GDM. It was revealed that no participant had clinical retinal changes. Furthermore, it was found that higher levels for the 1 h OGTT were associated with a drop in the CST and total thickness. Moreover, women who underwent CS had higher levels of total thickness. The superior-temporal retinal vein thickness was higher in women who underwent CS. In addition, higher levels of fasting OGTT were associated with a thinner inferior-temporal retinal artery. Interestingly, pregnant women with a previous history of miscarriage had lower macular density on FAF, as represented by lower values of integrated density and mean gray values. Furthermore, higher levels for the fasting OGTT were associated with higher values of integrated density. Moreover, higher dioptric powers of the spherical equivalent were associated with lower values of both the integrated density and mean gray value.

Diabetic retinopathy plays a major role in public health. It is considered the major cause of blindness in diabetic patients. Microvascular complications, including retinopathy, are divided into the proliferative stage, which contains neovascularization, and non-proliferative stage, which is characterized by the presence of micro aneurysms, retinal hemorrhages, and intraretinal microvascular abnormalities together with soft and hard exudates [10,11]. Therefore, early screening and detection help in preventing blindness, with special consideration of pregnant women, as several studies were conducted on them. Regarding chronic (pre-existing diabetes recognized during pregnancy) diabetes mellitus in pregnancy, Temple et al. conducted a study reflecting the relation between the duration of diabetic retinopathy type 1 in addition to the baseline background retinopathy changes at the beginning of pregnancy and diabetic progression. The findings suggest that both increase the progression; however, moderate–severe retinopathy changes play a stronger role than the duration [12]. They found a significant progression by the end of the second trimester [12]. Moloney and Drunry compared the progression of retinopathy in pregnant and non-pregnant women and the results revealed increases in background retinopathy along with the appearance of new onset retinal lesions [13]. However, despite an exclusive finding, fundus autofluorescence imaging was not performed [13]. Pregnancy itself, with the hormonal changes during it, can affect the blood flow and vessel integrity in the retina. Furthermore, the elevated metabolic demands and the fluctuation in blood pressure may contribute to retinal pathology [10,11,14,15,16]. It was reported that the rate of progression of diabetic retinopathy was twice as high in pregnant women with diabetes compared non-pregnant women [10]. The Diabetes Control and Complications Trial (DCCT) revealed that the odds of diabetic retinopathy progression in pregnant women were 1.63 in the intensively treated group and 2.48 in the regularly treated group compared to non-pregnant women [17]. Many reports showed that diabetic retinopathy progressed in the first and second trimesters, peaked at the end of the second trimester, and regressed in the third trimester [12,17,18,19]. The rate of development of diabetic retinopathy in eyes without previous diabetic retinopathy during pregnancy has been reported to be 15% [10,19]. It was higher in type 1 diabetes (15.8%) compared to type 2 diabetes (9.0%). The progression rate from the non-proliferative diabetic retinopathy stage to the proliferative diabetic retinopathy stage was 6.3% and the rate of progression was similar for both type 1 and type 2 diabetes [10,19].

On the other hand, the impact of GDM on the retina is less well understood, primarily because GDM is usually transient and is typically diagnosed later in pregnancy. GDM potentiates the risk of short-term antepartum complications such as preeclampsia and long-term postpartum complications such as dyslipidemia, hypertension, obesity, and type 2 diabetes mellitus, with its subsequent generalized endothelial and small-vessel vasculopathy [20,21,22,23,24]. Type 2 diabetes mellitus is associated with retinal arteriolar narrowing, greater retinal vascular tortuosity, retinal venular widening, and retinal vascular capillary bed reduction [25,26]. Whether these endothelial cell dysfunctions and small-vessel vasculopathy are present in GDM has not been well investigated. Accordingly, Liu and Wang conducted a study evaluating the microvasculature in GDM women compared to non-GDM pregnant women and non-pregnant women utilizing OCT–angiography [27]. They revealed that there was a significant reduction in the vascular density in superficial capillary layer with an increase in the deep capillary layer in the GDM and in the non-GDM pregnant women. However, more capillary “dropout” changes were detected in the GDM group. Unexpectedly, the GDM group revealed the improvement in the central macular thickness thinning and foveal avascular zone enlargement during pregnancy [27]. Li et al. conducted an interesting study regarding the effect of GDM on the retinal microvasculature using retinal photographs with a subsequent computer analyzer [26]. They demonstrated that women with GDM had narrower arteriolar caliber, reduced arteriolar fractal dimension, and larger arteriolar branching angle than women without GDM [26]. Another study conducted by the same investigators aimed to assess the association between the second-trimester retinal microvasculature and the 5-year metabolic syndrome incidence in women with GDM using retinal photographs [28]. Each 10-micron widening of retinal venular caliber was associated with an increased relative risk of 1.6 in the incidence of metabolic syndrome. The retinal venular caliber mildly increased the prediction of 5-year maternal metabolic syndrome by 1.8% [28].

Retinal macular and vascular imaging used to assess the retinal status in women with GDM is now a non-invasive [25,29]. FAF is a non-invasive imaging technique that detects fluorophores, naturally occurring molecules that absorb and emit light of specified wavelengths [30]. There are two main technologies that allow FAF acquisition, the conventional fundus camera and the confocal scanning laser ophthalmoscope [30,31,32]. As the excitation and emission spectra of retinal fluorophores usually overlay in part, both technologies select certain wavelengths for excitation and emission that avoid the overlay and allow for clear differentiation of the two [32]. The conventional method utilizes barrier filters for excitation and emission to select the required wavelength, and subsequently, the entire fundus is excited at once. This results in a phenomenon called “pseudoautofluorescence”, which is defined as scattered light originating from other sources mainly the crystalline lens [31]. Most available conventional devices use excitation wavelengths in the range of 510–580 nm and detection wavelengths in the range of 615–735 nm [31]. In contrast, the confocal scanning laser ophthalmoscope uses a monochromatic laser beam and a point detector that have the same focal plane. Accordingly, the scattered light is minimum [31]. A study by Yoshitake et al. investigated and quantified the signal intensity of FAF and evaluated its association with visual function and OCT findings. They revealed that eyes with diabetic macular edema had lower FAF signal intensity levels in the parafoveal subfields compared with eyes without diabetic macular edema. The FAF intensity in the parafoveal subfields was negatively associated with the visual acuity and the retinal thickness in the corresponding subfields [33]. Another study was conducted by Vujosevic et al. to quantitatively evaluate color FAF in patients with diabetes mellitus and to correlate these data with different stages of retinal disease severity using a 450 nm FAF device. Higher levels of intensity were found in patients affected by diabetes mellitus compared to healthy, non-diabetic subjects, and in patients with diabetic macular edema compared to patients without diabetic macular edema [34]. An interesting study was conducted by Kimura et al. to quantify FAF in laser photocoagulation scars for diabetic retinopathy and to compare the conventional and short-pulse laser. The average luminosity ratio of laser scars at 1, 3, 6, 12, and 18 months was higher for short-pulse laser compared to the conventional laser. These findings suggest that the short-pulse laser displayed delayed hypo-FAF progression [35]. Similar to these retinal imaging studies, we postulate that investigations performed concerning microvascular changes in GDM may provide valuable insights into such pregnancy complications. It is suggested that FAF imaging may be useful for detecting early retinal changes in patients without diabetic retinopathy. As a subclinical indicator for further retinal pathology, the lipofuscin granule in diabetic retinopathy might accumulate in the microglial cells rather than RPE cells, so the increase in FAF macular density might reveal deteriorated function of the neurosensory retina.

Regarding cataract in pregnancy, we have reported a high incidence of cataract (13.2%). GDM has the effect of cataract formation both during and after pregnancy. During pregnancy, high levels of glucose converted into sorbitol can accumulate in the lens and affect the integrity early on, even if the exposure is short [36]. Auger et al. demonstrated that GDM may be an independent risk factor for cataract, even among women who never develop type 2 diabetes, and most of these cases of cataract are mild [36].

Our study is not without limitations and has several limitations. Firstly, the small number of participants enrolled in our study may restrict the generalizability of our findings. Therefore, attention should be paid when extrapolating the results. Secondly, the lack of a control group may limit the comparability of the results with another group, with a subsequent deficit in the generalizability of the findings. Thirdly, many other investigations with interesting findings, such OCT–angiography, were not performed due to the unavailability of these resources at our institution. Lastly, certain confounder factors, such as the variability in the treatment adherence, were not investigated.

In conclusion, certain microvascular changes could reveal the liability of women with GDM for other long-term complications. Poorer control of the GDM, with higher levels at presentation, may be associated with more severe microvascular changes. Certain risk factors, such as a history of miscarriage or the mode of delivery, may warrant further evaluation. Larger studies with more investigations may reveal further findings. Regular ophthalmic screening for women with GDM is warranted at the diagnosis of GDM, antepartum, and after delivery. Women at risk should even be screened more frequently.

## Figures and Tables

**Table 1:** General demographical and clinical characteristics.

Variables	Number *	Percentage (%)
	Mean ± SEM	
Mother’s age (years)	32.7 ± 0.7	
Gestation age at delivery (weeks)	38.22 ± 0.2	
Gestational age at diagnosis of GDM (weeks)	30.3 ± 0.3	
Gestational age at ophthalmic examination (weeks)	30.9 ± 0.7	
Gestational parameters		
Gravity	4.0 ± 0.2	
Miscarriages	0.88 ± 0.1	
Method of GDM treatment (out of 34 participants)		
Diet only	5	14.7
OHGA-based regimen	12	35.3
Insulin-based regimen	17	50.0
Mean values of OGTT (mmol/L):		
Fasting OGTT	5.49 ± 0.1	
One-hour OGTT	9.81 ± 0.3	
Two-hour OGTT	8.38 ± 0.2	
Mode of delivery (out of 34 participants)		
CS	28	82.4
NVD	6	17.6
Fetal weight (grams)	2763.7 ± 0.1	
Ophthalmic examination findings (out of 68)		
Cataract	9	13.2
Corneal and anterior segment pathology	2	2.9
Posterior segment pathology	0	0.0
Optical parameters		
Uncorrected visual acuity (LogMAR)	0.81 ± 0.03	
Best-corrected visual acuity (LogMAR)	0.97 ± 0.01	
IOP (mmHg)	14.9 ± 0.2	
Spherical equivalent (Diopter)	−1.55 ± 0.2	
OCT parameters		
CST (micron)	259.7 ± 5.5	
Foveal minimum (micron)	200.7 ± 6.1	
Total thickness (micron)	2692.6 ± 32.8	
Total volume (mm^3^)	8.3 ± 0.1	
Retinal vascular thickness through autofluorescence images (mm)		
Superior-temporal retinal artery	0.1217 ± 0.003	
Superior-temporal retinal vein	0.1507 ± 0.004	
Inferior-temporal retinal artery	0.1286 ± 0.003	
Inferior-temporal retinal vein	0.1535 ± 0.004	
Macular density parameters through autofluorescence images		
Total area of investigation (mm^2^)	28.06 ± 0.01	
Mean gray value (pixel)	135.54 ± 3.1	
Maximum gray value (pixel)	231.89 ± 3.9	
Minimum gray value (pixel)	37.82 ± 4.7	
Integrated density (pixel/mm)	3789.47 ± 86.4	
Raw integrated density (pixel/mm)	7,702,068.87 ± 1784.88	

Abbreviations: GDM: gestational diabetes mellitus; OGTT: oral glucose tolerance test; OHGA: oral hypoglycemic agent; CS: cesarean section; NVD: normal vaginal delivery; OCT: optical coherence tomography; CST: central subfield thickness; SEM: standard error of mean. * N = 34.

**Table 2:** Factors affecting the OCT parameters.

Variables	Mean ± SEM * or B Regression Coefficient ± SEM **			
	CST (Micron)	*p*-Value	Total Thickness (Micron)	*p*-Value
Mother’s age (years) **	−0.33 ± 0.9	0.73	−2.72 ± 5.4	0.62
Gestation age at delivery (weeks) **	−0.28 ± 2.1	0.89	−10.19 ± 7.0	0.43
Gestational age at ophthalmic examination (weeks) **	−2.32 ± 1.2	0.07	−4.47 ± 8.0	0.56
Gestational parameters **				
Gravity	−3.27 ± 2.3	0.17	−9.74 ± 14.1	0.49
Miscarriages	0.39 ± 5.4	0.94	0.40 ± 3.1	0.99
Method of GDM treatment *				
Diet only	249.75 ± 5.5		2681.36 ± 89.3	
OHGA-based regimen	280.35 ± 11.5	0.43	2757.00 ± 187.8	0.58
Insulin-based regimen	252.70 ± 19.1		2665.56 ± 196.7	
Mean values of OGTT (mmol/L) **				
Fasting OGTT	−12.02 ± 7.8	0.13	−75.96 ± 14.5	0.10
One-hour OGTT	−7.28 ± 3.1	0.024	−54.57 ± 17.2	0.03
Two-hour OGTT	−0.37 ± 3.9	0.93	10.927 ± 22.9	0.63
Mode of delivery *				
CS	263.57 ± 5.7	0.06	2763.47 ± 236.4	0.001
NVD	243.40 ± 4.6		2395.10 ± 346.1	
Fetal weight (grams) **	−7.10 ± 6.1	0.58	−97.45 ± 41.2	0.18
Optical parameters **				
Best-corrected visual acuity (LogMAR)	−277.27 ± 11.8	0.018	−228.65 ± 61.5	0.74
IOP (mmHg)	1.60 ± 3.2	0.62	−12.08 ± 11.4	0.30
Spherical equivalent (Diopter)	−12.99 ± 7.9	0.11	−97.048 ± 14.6	0.032

Abbreviations: GDM: gestational diabetes mellitus; OGTT: oral glucose tolerance test; OHGA: oral hypoglycemic agent; CS: cesarean section; NVD: normal vaginal delivery; CST: central subfield thickness; SEM: standard error of mean. * ANOVA test, ** linear regression analysis.

**Table 3:** Factors affecting the thickness of the retinal vasculature.

Variables	Mean ± SEM * or B Regression Coefficient ± SEM **							
	Superior-Temporal Retinal Artery Thickness (mm)	*p*-Value	Superior-Temporal Retinal Vein Thickness (mm)	*p*-Value	Inferior-Temporal Retinal Artery Thickness (mm)	*p*-Value	Inferior-Temporal Retinal Vein Thickness (mm)	*p*-Value
Mother’s age (years) **	0.001 ± 0.001	0.31	0.001 ± 0.001	0.30	0.0001 ± 0.001	0.72	0.0001 ± 0.001	0.64
Gestation age at delivery (weeks) **	0.002 ± 0.001	0.74	0.003 ± 0.002	0.11	0.0005 ± 0.001	0.74	0.002 ± 0.002	0.29
Gestational age at ophthalmic examination (weeks) **	0.0001 ± 0.002	0.83	0.003 ± 0.002	0.08	0.001 ± 0.001	0.26	0.001 ± 0.003	0.71
Gestational parameters **								
Gravity	0.001 ± 0.001	0.57	0.0001 ± 0.002	0.90	0.003 ± 0.001	0.72	0.001 ± 0.002	0.51
Miscarriages	−0.003 ± 0.003	0.42	−0.007 ± 0.004	0.09	−0.003 ± 0.004	0.42	−0.004 ± 0.005	0.43
Method of GDM treatment *								
Diet only	0.126 ± 0.02		0.143 ± 0.03		0.133 ± 0.01		0.160 ± 0.04	
OHGA-based regimen	0.115 ± 0.02	0.41	0.150 ± 0.02	0.65	0.130 ± 0.003	0.75	0.153 ± 0.001	0.77
Insulin-based regimen	0.123 ± 0.03		0.154 ± 0.03		0.126 ± 0.004		0.151 ± 0.005	
Mean values of OGTT (mmol/L) **								
Fasting OGTT	−0.004 ± 0.004	0.37	−0.005 ± 0.006	0.34	−0.01 ± 0.004	0.01	−0.009 ± 0.006	0.11
One-hour OGTT	0.001 ± 0.002	0.60	0.0001 ± 0.003	0.91	−0.002 ± 0.002	0.39	−0.002 ± 0.003	0.52
Two-hour OGTT	0.003 ± 0.002	0.12	0.0001 ± 0.003	0.89	0.0001 ± 0.002	0.85	0.001 ± 0.003	0.72
Mode of delivery *								
CS	0.126 ± 0.02	0.06	0.155 ± 0.02	0.018	0.129 ± 0.003	0.83	0.156 ± 0.005	0.15
NVD	0.107 ± 0.01		0.128 ± 0.03		0.127 ± 0.008		0.139 ± 0.007	
Fetal weight (grams) **	−0.004 ± 0.006	0.55	−0.006 ± 0.008	0.50	−0.004 ± 0.005	0.61	−0.008 ± 0.007	0.27
Optical parameters **								
Best-corrected visual acuity (LogMAR)	−0.11 ± 0.07	0.17	−0.007 ± 0.09	0.94	−0.16 ± 0.07	0.03	−0.233 ± 0.09	0.02
IOP (mmHg)	−0.003 ± 0.002	0.24	−0.005 ± 0.003	0.07	0.001 ± 0.002	0.75	0.003 ± 0.003	0.35
Spherical equivalent (Diopter)	−0.005 ± 0.003	0.17	−0.007 ± 0.003	0.06	0.001 ± 0.002	0.69	0.005 ± 0.003	0.98

Abbreviations: GDM: gestational diabetes mellitus; OGTT: oral glucose tolerance test; OHGA: oral hypoglycemic agent; CS: cesarean section; NVD: normal vaginal delivery; SEM: standard error of mean. * ANOVA test, ** linear regression analysis.

**Table 4:** Factors affecting the density parameters of the autofluorescence images.

Variables	Mean ± SEM * or B Regression Coefficient ± SEM **			
	Mean Gray Value (pixel)	*p*-Value	Integrated Density (pixel/mm)	*p*-Value
Mother’s age (years) **	0.045 ± 0.05	0.40	13.04 ± 11.5	0.38
Gestation age at delivery (weeks) **	2.19 ± 1.2	0.07	57.22 ± 33.1	0.09
Gestational age at ophthalmic examination (weeks) **	−1.73 ± 1.5	0.29	−48.73 ± 43.4	0.28
Gestational parameters **				
Gravity	0.22 ± 1.3	0.87	13.26 ± 31.9	0.71
Miscarriages	−6.50 ± 3.1	0.042	−173.75 ± 58.7	0.049
Method of GDM treatment *				
Diet only	140.06 ± 6.0		3932.15 ± 169.1	
OHGA-based regimen	142.14 ± 4.9	0.28	3928.61 ± 125.1	0.37
Insulin-based regimen	131.12 ± 23.8		3681.34 ± 130.6	
Mean values of OGTT (mmol/L) **				
Fasting OGTT	7.29 ± 3.4	0.07	228.001 ± 89.4	0.04
One-hour OGTT	1.50 ± 2.1	0.49	50.25 ± 57.8	0.39
Two-hour OGTT	1.09 ± 2.1	0.61	28.58 ± 59.0	0.63
Mode of delivery *				
CS	136.63 ± 3.8	0.52	3815.45 ± 105.9	0.57
NVD	131.64 ± 4.1		3695.90 ± 116.1	
Fetal weight (grams) **	9.76 ± 6.1	0.16	264.16 ± 148.5	0.17
Optical parameters **				
Best corrected visual acuity (LogMAR)	35.89 ± 21.1	0.64	907.5 ± 1078.3	0.63
IOP (mmHg)	−0.71 ± 2.0	0.73	−30.3 ± 56.5	0.60
Spherical equivalent (Diopter)	−10.95 ± 2.9	0.002	−298.9 ± 81.3	0.001

Abbreviations: GDM: gestational diabetes mellitus; OGTT: oral glucose tolerance test; OHGA: oral hypoglycemic agent; CS: cesarean section; NVD: normal vaginal delivery; SEM: standard error of mean. * ANOVA test.** Linear regression analysis.

## Data Availability

Data generated from this study are available upon request from the corresponding author.

## References

[1] McIntyre H.D., Catalano P., Zhang C., Desoye G., Mathiesen E.R., Damm P. (2019). Gestational diabetes mellitus. Nat. Rev. Dis. Primers.

[2] Francis E.C., Powe C.E., Lowe W.L., White S.L., Scholtens D.M., Yang J., Zhu Y., Zhang C., Hivert M.-F., Kwak S.H. (2023). Refining the diagnosis of gestational diabetes mellitus: A systematic review and meta-analysis. Commun. Med..

[3] Lee K.W., Ching S.M., Ramachandran V., Yee A., Hoo F.K., Chia Y.C., Sulaiman W.A.W., Suppiah S., Mohamed M.H., Veettil S.K. (2018). Prevalence and risk factors of gestational diabetes mellitus in Asia: A systematic review and meta-analysis. BMC Pregnancy Childbirth.

[4] Vargas S.E.V., Chávez-González E.L. (2023). Obstetric-Neonatal Complications of Gestational Diabetes: A Systematic Review. Mex. J. Med. Res. ICSA.

[5] Alum E.U., Ugwu O.P., Obeagu E.I. (2024). Beyond Pregnancy: Understanding the Long-Term Implications of Gestational Diabetes Mellitus. INOSR Sci. Res..

[6] Beharier O., Sergienko R., Kessous R., Szaingurten-Solodkin I., Walfisch A., Shusterman E., Tsumi E., Sheiner E. (2017). Gestational diabetes mellitus is a significant risk factor for long-term ophthalmic morbidity. Arch. Gynecol. Obstet..

[7] Madike R., Cugati S., Qin Q., Chen C.J. (2024). Pregnancy and the eye: What do we need to watch out for? A review. Clin. Exp. Ophthalmol..

[8] Chandrasekaran P.R., Madanagopalan V.G., Narayanan R. (2021). Diabetic retinopathy in pregnancy—A review. Indian J. Ophthalmol..

[9] Angeli O., Hajdu D., Jeney A., Czifra B., Nagy B.V., Balazs T., Nemoda D.J., Somfai G.M., Nagy Z.Z., Peto T. (2023). Qualitative and quantitative comparison of two semi-manual retinal vascular density analyzing methods on optical coherence tomography angiography images of healthy individuals. Sci. Rep..

[10] Widyaputri F., Rogers S.L., Kandasamy R., Shub A., Symons R.C.A., Lim L.L. (2022). Global Estimates of Diabetic Retinopathy Prevalence and Progression in Pregnant Women with Preexisting Diabetes: A Systematic Review and Meta-analysis. JAMA Ophthalmol..

[11] Rosenn B., Miodovnik M., Kranias G., Khoury J., Combs C.A., Mimouni F., Siddiqi T.A., Lipman M.J. (1992). Progression of diabetic retinopathy in pregnancy: Association with hypertension in pregnancy. Am. J. Obstet. Gynecol..

[12] Temple R.C., Aldridge V.A., Sampson M.J., Greenwood R.H., Heyburn P.J., Glenn A. (2001). Impact of pregnancy on the progression of diabetic retinopathy in Type 1 diabetes. Diabet. Med. A J. Br. Diabet. Assoc..

[13] Moloney J.B., Drury M.I. (1982). The effect of pregnancy on the natural course of diabetic retinopathy. Am. J. Ophthalmol..

[14] Best R.M., Chakravarthy U. (1997). Diabetic retinopathy in pregnancy. Br. J. Ophthalmol..

[15] Gupte S., Venkataraman G., Shah A.S., Jamenis S., Rao C., Jangam S.M., Adki K.M., Swami O.C. (2023). Prevalence and outcomes of gestational diabetes mellitus in Indian women: Insights from a large real-world study over ten years at tertiary care research institute. Int. J. Diabetes Dev. Ctries..

[16] Schreiberová Z., Chrapek O., Šimičák J. (2020). Ocular Complications of Diabetes Mellitus in Pregnancy—Case Report. Ceska A Slov. Oftalmol. Cas. Ceske Oftalmol. Spol. A Slov. Oftalmol. Spol..

[17] Diabetes Control and Complications Trial Research Group (2000). Effect of pregnancy on microvascular complications in the diabetes control and complications trial. The Diabetes Control and Complications Trial Research Group. Diabetes Care.

[18] Venkatesh P., Makwana T., Takkar B., Sharma J.B., Gupta Y., Chawla R., Vohra R., Kriplani A., Tandon N., Venkatesh P. (2018). Prevalence, progression, and outcomes of diabetic retinopathy during pregnancy in Indian scenario. Indian J. Ophthalmol..

[19] Morrison J.L., Hodgson L.A., Lim L.L., Al-Qureshi S. (2016). Diabetic retinopathy in pregnancy: A review. Clin. Exp. Ophthalmol..

[20] Durnwald C. (2015). Gestational diabetes: Linking epidemiology, excessive gestational weight gain, adverse pregnancy outcomes, and future metabolic syndrome. Semin. Perinatol..

[21] Feig D.S., Zinman B., Wang X., Hux J.E. (2008). Risk of development of diabetes mellitus after diagnosis of gestational diabetes. CMAJ.

[22] Taylor R. (2013). Type 2 diabetes: Etiology and reversibility. Diabetes Care.

[23] Aldhahi W., Hamdy O. (2003). Adipokines, inflammation, and the endothelium in diabetes. Curr. Diab Rep..

[24] Bonetti P.O., Lerman L.O., Lerman A. (2003). Endothelial dysfunction: A marker of atherosclerotic risk. Arterioscler. Thromb. Vasc. Biol..

[25] Ikram M.K., Cheung C.Y., Lorenzi M., Klein R., Jones T.L., Wong T.Y. (2013). Retinal vascular caliber as a biomarker for diabetes microvascular complications. Diabetes Care.

[26] Li L.-J., Kramer M., Tapp R.J., Man R.E.K., Lek N., Cai S., Yap F., Gluckman P., Tan K.H., Chong Y.S. (2017). Gestational diabetes mellitus and retinal microvasculature. BMC Ophthalmol..

[27] Liu G., Wang F. (2021). Macular vascular changes in pregnant women with gestational diabetes mellitus by optical coherence tomography angiography. BMC Ophthalmol..

[28] Li L.-J., Tan K.H., Aris I.M., Man R.E.K., Gan A.T.L., Chong Y.S., Saw S.M., Gluckman P., Wong T.Y., Lamoureux E. (2018). Retinal vasculature and 5-year metabolic syndrome among women with gestational diabetes mellitus. Metabolism.

[29] Benitez-Aguirre P.Z., Sasongko M.B., Craig M.E., Jenkins A.J., Cusumano J., Cheung N., Wong T.Y., Donaghue K.C. (2012). Retinal vascular geometry predicts incident renal dysfunction in young people with type 1 diabetes. Diabetes Care.

[30] Yung M., Klufas M.A., Sarraf D. (2016). Clinical applications of fundus autofluorescence in retinal disease. Int. J. Retin. Vitr..

[31] Dumitrescu O.-M., Zemba M., Brănișteanu D.C., Pîrvulescu R.A., Radu M., Stanca H.T. (2024). Fundus Autofluorescence in Diabetic Retinopathy. J. Pers. Med..

[32] Calvo-Maroto A.M., Esteve-Taboada J.J., Domínguez-Vicent A., Pérez-Cambrodí R.J., Cerviño A. (2016). Confocal scanning laser ophthalmoscopy versus modified conventional fundus camera for fundus autofluorescence. Expert Rev. Med. Devices.

[33] Yoshitake S., Murakami T., Uji A., Unoki N., Dodo Y., Horii T., Yoshimura N. (2015). Clinical relevance of quantified fundus autofluorescence in diabetic macular oedema. Eye.

[34] Vaclavik V., Vujosevic S., Dandekar S.S., Bunce C., Peto T., Bird A.C. (2008). Autofluorescence imaging in age-related macular degeneration complicated by choroidal neovascularization: A prospective study. Ophthalmology.

[35] Kimura T., Ogura S., Yasukawa T., Nozaki M. (2023). Quantitative Evaluation of Fundus Autofluorescence in Laser Photocoagulation Scars for Diabetic Retinopathy: Conventional vs. Short-Pulse Laser. Life.

[36] Auger N., Tang T., Healy-Profitós J., Paradis G. (2017). Gestational diabetes and the long-term risk of cataract surgery: A longitudinal cohort study. J. Diabetes Complicat..

